# Computational identification and characterization of glioma candidate biomarkers through multi-omics integrative profiling

**DOI:** 10.1186/s13062-020-00264-5

**Published:** 2020-06-15

**Authors:** Lin Liu, Guangyu Wang, Liguo Wang, Chunlei Yu, Mengwei Li, Shuhui Song, Lili Hao, Lina Ma, Zhang Zhang

**Affiliations:** 1 China National Center for Bioinformation, Beijing, 100101 China; 2 National Genomics Data Center & CAS Key Laboratory of Genome Sciences and Information, Beijing Institute of Genomics, Chinese Academy of Sciences, Beijing, 100101 China; 3 University of Chinese Academy of Sciences, Beijing, 100101 China; 4 Present Address: The Methodist Hospital Research Institute, 6670 Bertner Ave, Houston, TX 77030 USA; 5 Division of Biomedical Statistics and Informatics, Mayo Clinic College of Medicine, Rochester, MN 55905 USA

**Keywords:** Glioma, Multi-omics, *PRKCG*, Biomarker, Cerebrospinal fluid

## Abstract

**Background:**

Glioma is one of the most common malignant brain tumors and exhibits low resection rate and high recurrence risk. Although a large number of glioma studies powered by high-throughput sequencing technologies have led to massive multi-omics datasets, there lacks of comprehensive integration of glioma datasets for uncovering candidate biomarker genes.

**Results:**

In this study, we collected a large-scale assemble of multi-omics multi-cohort datasets from worldwide public resources, involving a total of 16,939 samples across 19 independent studies. Through comprehensive molecular profiling across different datasets, we revealed that *PRKCG* (Protein Kinase C Gamma), a brain-specific gene detectable in cerebrospinal fluid, is closely associated with glioma. Specifically, it presents lower expression and higher methylation in glioma samples compared with normal samples. *PRKCG* expression/methylation change from high to low is indicative of glioma progression from low-grade to high-grade and high RNA expression is suggestive of good survival. Importantly, *PRKCG* in combination with *MGMT* is effective to predict survival outcomes in a more precise manner.

**Conclusions:**

*PRKCG* bears the great potential for glioma diagnosis, prognosis and therapy, and *PRKCG*-like genes may represent a set of important genes associated with different molecular mechanisms in glioma tumorigenesis. Our study indicates the importance of computational integrative multi-omics data analysis and represents a data-driven scheme toward precision tumor subtyping and accurate personalized healthcare.

## Background

Glioma, one of the serious central nervous system (CNS) tumors, represents ~ 80% of malignant brain tumors [1, 2] and exhibits low resection rate and high recurrence risk [3]. Since tumor classification benefits accurate diagnosis and facilitates precise treatment, gliomas can be classified, according to the histologic grading schemes, into LGG (astrocytoma, oligodendroglioma and oligoastrocytoma) and GBM (glioblastoma multiforme) [4]. Therefore, identification of reliable molecular biomarkers for precise classification of different-grade gliomas is crucial to aid tumor diagnosis, establish appropriate therapies, recognize prognostic outcome and predict therapeutic response [5].

Powered by high-throughput sequencing technologies, a set of molecular biomarkers have been discovered from different omics levels to assist glioma diagnosis and treatment [6, 7]. Among them, isocitrate dehydrogenase (*IDH*) mutation and 1p/19q co-deletion (codel) are two most important genetic events for glioma grading [8–10]. Patients with *IDH* mutation (*IDH*-mut) have longer survival than those with *IDH* wild-type (*IDH*-WT) [11, 12]. And the 1p/19q codel is a distinctive feature of oligodendroglioma [13, 14]. Furthermore, based on these two genetic alterations, accumulated evidence suggested that gliomas can be divided into three subtypes (*IDH*-mut & 1p/19q codel, *IDH*-mut & 1p/19q non-codel, and *IDH*-WT & 1p/19q non-codel), which are associated with diverse clinical outcomes [15]. Accordingly, in 2016, the World Health Organization (WHO), in light of both histology and significant genetic events (mainly by *IDH* and 1p/19q), divided gliomas into five categories [16, 17], including three LGGs (diffuse astrocytoma, *IDH*-mut & 1p/19q non-codel; oligodendroglioma, *IDH*-mut & 1p/19q codel; diffuse astrocytoma, *IDH*-WT & 1p/19q non-codel) and two GBMs (*IDH*-mut; *IDH*-WT). Meanwhile, molecular markers at the transcriptome level have also been identified [18–21]; for example, an overexpression of epidermal growth factor receptor variant III (*EGFRvIII*) has been reported to associate with malignant progression of GBM [22–24]. In addition, epigenetic modifications are also implicated in glioma [25–29]. One classical biomarker is O6-methylguanine-DNA-methyltransferase (*MGMT*) [30]; patients with methylated *MGMT* promoter have better clinical outcomes and are more sensitive to the alkylating chemotherapy than those without methylated *MGMT* promoter [31–33].

Nowadays, there is an increasing number of high-throughput studies for better understanding of glioma tumorigenesis [34–38], resulting in massive multi-omics datasets generated from different projects and laboratories throughout the world. However, there lacks of comprehensive integration of glioma datasets for computationally identifying and characterizing candidate biomarkers. To this end, we collected a large-scale assemble of multi-omics multi-cohort datasets from worldwide public resources and detected candidate biomarker genes through comprehensive integrative molecular profiling on multiple independent datasets. We revealed that *PRKCG*, a gene specifically expressed in brain and detectable in cerebrospinal fluid (CSF), is closely associated with glioma, indicative of a potential biomarker for glioma diagnosis, prognosis and treatment prediction.

## Materials and methods

### Data collection

In this study, we collected a comprehensive assemble of multi-omics datasets (including genomics, transcriptomics, DNA methylomics and proteomics) from The Cancer Genome Atlas (TCGA, https://portal.gdc.cancer.gov/) [35], Genotype-Tissue Expression Portal (GTEx, https://gtexportal.org/home/) [39], Gene Expression Omnibus (GEO, https://www.ncbi.nlm.nih.gov/geo), Ivy Glioblastoma Atlas Project (Ivy GAP, http://glioblastoma.alleninstitute.org) [40] and Chinese Glioma Genome Atlas (CGGA, http://www.cgga.org.cn) [41, 42]. Particularly, discovery datasets were derived from TCGA, GTEx and large cohort studies in GEO (GSE83710, GSE16011 and GSE36278 for protein, expression and methylation, respectively). As a result, a total of five discovery datasets and 14 validation datasets were obtained. For convenience, each dataset collected in this study is assigned a unique accession number with the format: [D/V][*i*]-[TCGA/GTEx/GEO/CGGA/Ivy GAP]-[E/V/P/M], where D/V in the first bracket represents the dataset for discovery or validation, *i* in the second bracket indicates the dataset number, the third bracket shows the data source (as mentioned above), and the last bracket indicates the data type, namely, E for RNA expression, V for CNV, P for protein expression and M for DNA methylation, respectively. The detailed information about all collected datasets was tabulated in Table 1.

**Table 1:** Summary of multi-omics multi-cohort glioma datasets

Category	Accession number	Source	Omics type	# Samples	# Population country/race	Reference
**Discovery**	D1-GTEx-E	GTEx	Expression (RNA-Seq)	11,688	mostly white	[39]
	D2-GSE83710-P	GSE83710	Protein	133	Japan	[43]
	D3-GSE16011-E	GSE16011	Expression (Microarray)	284	Netherlands	[44]
	D4-TCGA-V	TCGA	CNV	1018	mostly white	[35]
	D4-TCGA-E	TCGA	Expression (RNA-Seq)	607	mostly white	[35]
	D4-TCGA-M	TCGA	Methylation (27 K + 450 K)	862	mostly white	[35]
	D4-TCGA-M (TMZ treatment)	TCGA	Methylation (27 K + 450 K)	228	mostly white	[35]
	D5-GSE36278-M	GSE36278	Methylation (450 K)	142	Germany	[45]
**Validation**	V1-GSE4290-E	GSE4290	Expression (Microarray)	180	USA	[46]
	V2-GSE50161-E	GSE50161	Expression (Microarray)	47	USA	[47]
	V3-GSE59612-E	GSE59612	Expression (RNA-Seq)	92	USA	[48]
	V4-GSE111260-E	GSE111260	Expression (Microarray)	70	Norway	–
	V5-GSE2223-E	GSE2223	Expression (Microarray)	54	USA	[49, 50]
	V6-Ivy GAP-E	Ivy GAP	Expression (RNA-Seq)	122	unknown	[40]
	V7-CGGA-E	CGGA	Expression (Microarray)	301	China	[42, 51]
	V8-GSE50923-M	GSE50923	Methylation (27 K)	78	USA	[52]
	V9-GSE61160-M	GSE61160	Methylation (450 K)	51	Spain	[53]
	V10-CGGA-M	CGGA	Methylation (27 K)	159	China	[54]
	V11-TCGA-M	TCGA	Methylation (WGBS)	6	white	–
	V12-CGGA-E	CGGA	Expression (RNA-Seq)	310	China	[41]
	V13-CGGA-E	CGGA	Expression (RNA-Seq)	667	China	–
	V14-GSE60274-M	GSE60274	Methylation (450 K)	68	Switzerland	[55]

*CGGA* Chinese Glioma Genome Atlas, http://www.cgga.org.cn

*GEO* Gene Expression Omnibus, https://www.ncbi.nlm.nih.gov/geo/

*GTEx* Genotype-Tissue Expression, https://www.gtexportal.org

*TCGA* The Cancer Genome Atlas, https://portal.gdc.cancer.gov

*Ivy GAP* Ivy Glioblastoma Atlas Project, http://glioblastoma.alleninstitute.org

### Identification of brain-specific genes

To identify brain-specific genes, we used the RNA-Seq dataset from GTEx (2016-01-15; v7) [39], which contains 11,688 samples across 53 tissue sites of 714 donors. Considering that several tissues have multiple different sites, gene expression levels were averaged over sites that are from the same tissue. To reduce background noise, genes with maximal expression levels smaller than 10 TPM (Transcripts Per Million) were removed. Finally, we obtained a total of 15,176 gene expression profiles across 30 tissues (Additional file 1: Table S1).

Based on the expression levels across 30 tissues, we calculated the tissue specificity index τ [56] for each gene to identify tissue-specific genes. τ is valued between 0 and 1, where 0 represents housekeeping genes that are consistently expressed in different tissues, and 1 indicates tissue-specific genes that are exclusively expressed in only one tissue [56]. In this study, brain-specific genes were defined as those genes that are maximally expressed in the brain with τ > 0.9. As a consequence, a list of the top 100 brain-specific genes ranked by the τ index were obtained for further analysis (Additional file 1: Table S2).

### Sample classification

To comprehensively study the potential of *PRKCG* in glioma diagnosis, we compared the molecular profiles between normal and glioma samples, between LGG and GBM samples, between primary GBM (pGBM) and recurrent GBM (rGBM) samples, and between glioma samples with different anatomic features. We collected 122 GBM samples from the Ivy GAP database [40] and grouped them according to their anatomic regions, namely, leading edge (LE, the ratio of tumor/normal cells is about 1–3/100), infiltrating tumor (IT, the ratio of tumor/normal cells is about 10–20/100), cellular tumor (CT, the ratio of tumor/normal cells is about 100/1 to 500/1), pseudo-palisading cells around necrosis (PAN, the narrow boundary of cells along the dead tissue), and microvascular proliferation (MVP, two or more blood vessels sharing a common vessel wall of endothelial).

We investigated the prognostic role of *PRKCG* by dividing samples into subgroups based on *PRKCG*’s expression level within all glioma samples and also within LGG and GBM samples, respectively. When exploring the predictive role of *PRKCG*, we obtained DNA methylation status (methylated and unmethylated) directly from the original study [35], which was defined based on the beta value cutoff 0.3.

### Identification of *PRKCG*-like genes

Genes that satisfy the following criteria were regarded as *PRKCG*-like genes: (1) Higher methylation level of at least one CpG site (promoter region) in glioma samples than normal samples; (2) Higher DNA methylation level in LGG samples than GBM samples; (3) Higher expression level in LGG samples than GBM samples; and (4) Lower expression level in glioma samples than normal samples. As a result, we obtained a total of 542 *PRKCG*-like genes, which were further divided into two groups according to their correlations between gene expression and methylation, namely, 114 genes with negative correlation and 297 genes with positive correlation.

### Statistical analysis

All statistical analyses were performed using R version 3.3.2. The Wilcoxon test was used for the analysis of the difference in gene expression/methylation between tumor and normal samples, and between different glioma subtypes. The statistical significance levels were coded by *ns* (not significant) *p* > 0.05, * *p* < 0.05, ** *p* < 0.01 and *** *p* < 0.001. We performed the survival analysis using the Kaplan-Meier method and estimated the statistical difference using the log-rank test.

## Results and discussion

### *PRKCG* is a brain-specific gene and detectable in cerebrospinal fluid

**Fig. 1:**
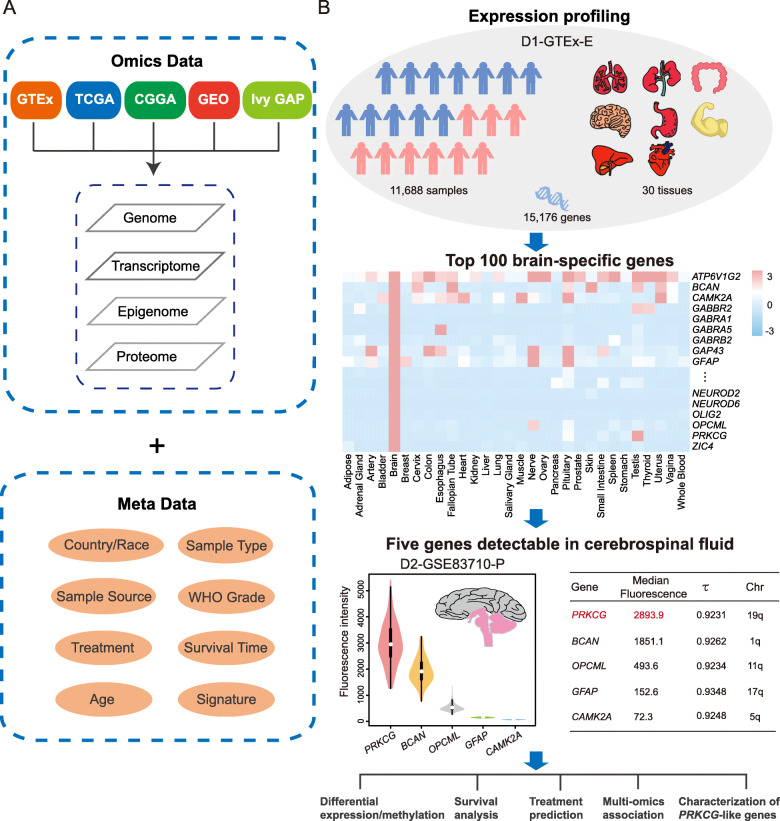
Datasets and bioinformatic analysis workflow. **a** A comprehensive assemble of multi-omics datasets and their corresponding meta data were integrated from GTEx, TCGA, CGGA, GEO and Ivy GAP. **b** An integrative analysis workflow was adopted, including detection of brain-specific genes, identification of CSF-detectable genes, ranking of candidate genes in light of protein fluorescence. A series of bioinformatic analyses were performed, including differential expression/methylation analysis, survival analysis, treatment prediction, multi-omics association and characterization of *PRKCG*-like genes

Tissue-specific genes are believed to be crucial for identifying potential biomarkers with high specificity [57–61]. To identify candidate genes with brain specificity, we integrated expression data from GTEx (D1-GTEx-E) [39], explored all genes’ expression profiles and their tissue specificity, and identified a list of top 100 brain-specific genes (Additional file 1: Table S2). To achieve the detectability in the periphery, we assembled a total of 1126 CSF-detectable proteins from GEO (D2-GSE83710-P) [43], due to the critical significance of CSF as a feasible means to detect genes expressed in human CNS [62, 63]. After integrating brain-specific genes with CSF proteins, we revealed that there are five brain-specific proteins that can be detected in CSF (Fig. 1 and Additional file 2: Fig. S1), in terms of fluorescence intensity from high to low, namely, *PRKCG* (protein kinase C gamma)*, BCAN* (brevican), *OPCML* (opioid binding protein/cell adhesion molecule like), *GFAP* (glial fibrillary acidic protein) and *CAMK2A* (calcium/calmodulin dependent protein kinase II alpha), which are diversely expressed in different brain regions (Additional file 3: Fig. S2). Specifically, *BCAN*, a member of the lectican family of chondroitin sulfate proteoglycans, is highly expressed in glioma and may promote cell motility of brain tumor cells [64, 65]. In addition, the fusion event between *BCAN* and *NTRK1* (*BCAN*-*NTRK1*) is a potential glioma driver and therapeutic target [66]. *OPCML* encodes a member of the IgLON subfamily in the immunoglobulin proteins and is down-regulated in gliomas and other brain tumors [67, 68]. *GFAP*, encoding one of the major intermediate filament proteins of mature astrocytes [69], can be used to assess the differentiation state of astrocytoma [70]. *CAMK2A* is a calcium calmodulin-dependent protein kinase and reduced expression of *CAMK2A* is associated with better survival in GBM [71, 72].

Remarkably, *PRKCG*, a member of protein kinase C (PKC) family located in 19q, exhibits higher fluorescence intensity than the other four genes (Fig. 1 and Additional file 2: Fig. S1). The expression profile of *PRKCG* across multiple brain developmental stages reveals that its expression is extremely lower in the prenatal stages, but dramatically increases in the infancy stages and is stabilized in the latter stages according to GenTree (Additional file 4: Fig. S3) [73]. Previous studies have documented that unlike other PKC family members that are expressed in many tissues aside from brain, *PRKCG* is brain-specifically expressed [74] and that mutations in *PRKCG* are associated with spinocerebellar ataxia [75, 76]. Additionally, it has been reported that PKC signaling pathways contribute to the aggressive behavior of glioma cells [77] and atypical PKC isozymes are fundamental regulators of tumorigenesis [78]. To our knowledge, several genes in 19q are closely associated with glioma (e.g., *TTYH1*(tweety family member 1), *UBE2S* (ubiquitin conjugating enzyme E2 S) [79, 80]). However, the potential role of *PRKCG* in glioma remains unknown, and therefore, comprehensive molecular characterization of *PRKCG* across multi-omics glioma datasets is highly desirable.

### *PRKCG* is significantly differentially expressed among normal, LGG and GBM samples

**Fig. 2:**
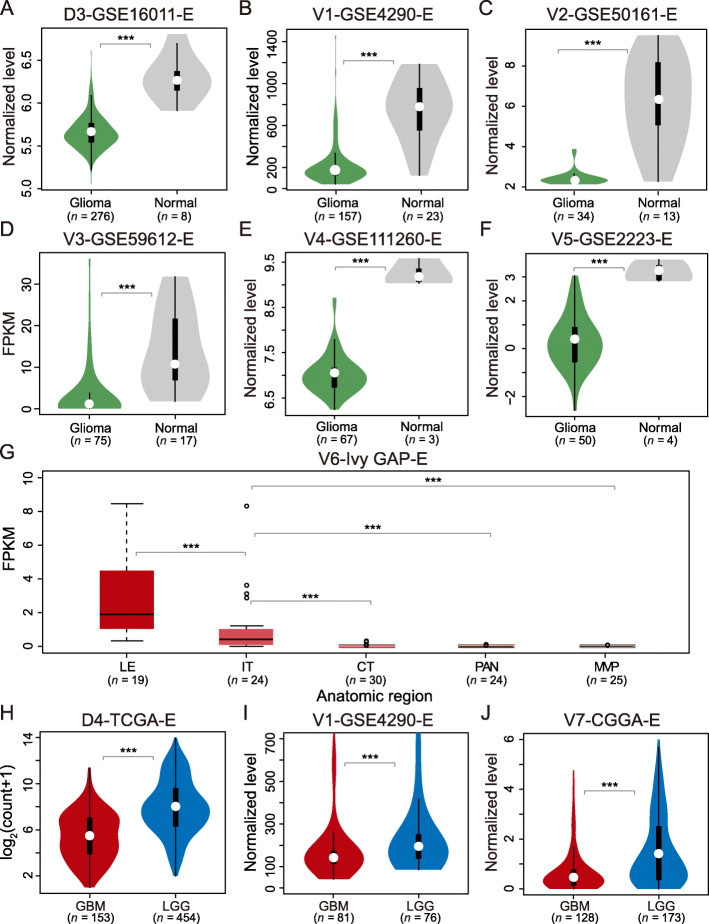
Expression profiles of *PRKCG* across normal, LGG and GBM samples. *PRKCG* expression profiles were compared between glioma and normal samples (D3-GSE16011-E in panel **a** [RMA normalized], V1-GSE4290-E in panel **b** [MAS5 normalized], V2-GSE50161-E in panel **c**[gcRMA normalized], V3-GSE59612-E in panel **d**, V4-GSE111260-E in panel **e** [RMA normalized], V5-GSE2223-E in panel **f** [Lowess normalized]), between different anatomic regions (V6-Ivy GAP-E in panel **g**), and between GBM and LGG samples (D4-TCGA-E in panel **h**, V1-GSE4290-E in panel **i** [MAS5 normalized] and V7-CGGA-E in panel **j** [Lowess normalized]). All the normalization methods labeled above were derived from and detailed in their corresponding publications, and all these datasets were made publicly accessible at ftp://download.big.ac.cn/glioma_data/. The Wilcoxon tests were performed and the statistical significance levels were coded by: *ns p* > 0.05, * *p* < 0.05, ** *p* < 0.01 and *** *p* < 0.001

We first investigated the expression pattern of *PRKCG* among normal, LGG and GBM samples by using multiple discovery and validation datasets. We found that *PRKCG* expression is significantly reduced in gliomas by contrast to normal samples (Fig. 2a-f; *p-*value < 0.01, Wilcoxon test). Furthermore, we discovered that *PRKCG* shows significantly different expression profiles among different anatomic regions (Fig. 2g; *p-*value < 0.01, Wilcoxon test). Strikingly, *PRKCG* expression is highest in LE (the outermost boundary of the tumor), decreased in IT (the intermediate zone between the LE and the serious CT regions), and lowest in the serious regions (CT, PAN and MVP) (see details in Materials and Methods). Consistently, comparison between different-grade gliomas showed that *PRKCG* expression is significantly lower in GBM samples than LGG samples (Fig. 2h-j; *p-*value < 0.01, Wilcoxon test). We further investigated its expression across pan-cancer samples. Although it has been documented that *PRKCG* is up-regulated in colon cancer [81], the up-regulation in colon cancer is extremely lower by comparison with glioma (LGG and GBM) (Additional file 5: Fig. S4). Taken together, these results presumably suggest that *PRKCG* is closely associated with glioma and its reduced expression is coupled with glioma progression (Fig. 2), highlighting its possible potential for glioma diagnosis.

### *PRKCG* expression is highly sensitive to survival

**Fig. 3:**
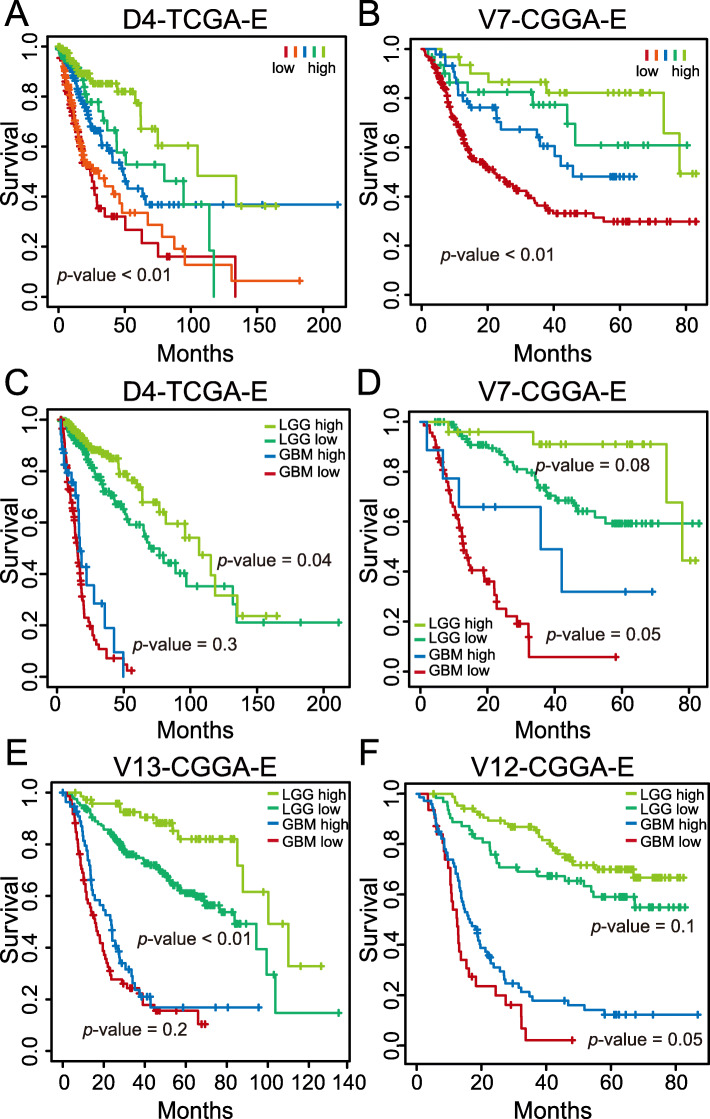
Expression profiles of *PRKCG* associated with survival. Glioma samples were divided into different groups based on *PRKCG* expression (panels **a** and **b**). LGG and GBM samples were divided into two groups with high expression and low expression, respectively (panels **c** to **f**). All these datasets can be publicly accessible at ftp://download.big.ac.cn/glioma_data/. The log-rank tests were used to examine the statistical significance between different survival curves

*PRKCG* expression change from high to low is indicative of progression from normal to glioma and from LGG to GBM (Fig. 2), implying that *PRKCG* expression is significantly associated with glioma progression. Importantly, we observed that *PRKCG* expression is significantly associated with survival rate, which is testified by multiple independent datasets (Fig. 3). Specifically, higher expression of *PRKCG* is indicative of longer overall survival in all glioma samples (Fig. 3a-b; *p*-value < 0.01, log-rank test). When separating LGG samples from GBM samples, it is consistently observed that higher expression, albeit not statistically significant in all examined datasets, tends to have longer overall survival in both LGG and GBM samples (Fig. 3c-f). Obviously, *PRKCG* expression has the potential capability to differentiate samples with diverse survival states, which would be of critical significance for accurate glioma subtyping, better therapeutic decisions and precision healthcare.

### *PRKCG* is significantly differentially methylated among normal, LGG and GBM samples

**Fig. 4:**
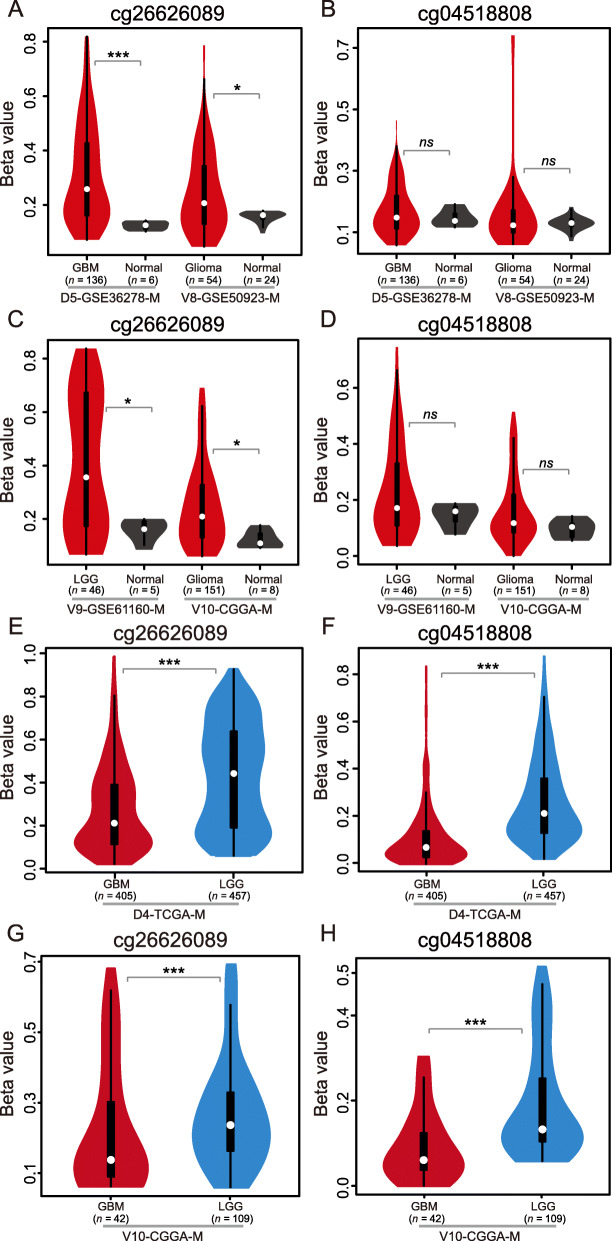
DNA methylation profiles across normal, LGG and GBM samples. *PRKCG* methylation profiles were compared between GBM and normal samples (panels **a** to **d**), and between LGG and GBM samples (panels **e** to **h**). All these datasets can be publicly accessible at ftp://download.big.ac.cn/glioma_data/. The Wilcoxon tests were used and their statistical significance levels were coded by: *ns p* > 0.05, * *p* < 0.05, ** *p* < 0.01 and *** *p* < 0.001

Since *PRKCG* harbors two CpG sites (namely, cg26626089 and cg04518808) that are located in the promoter region and covered in both HumanMethylation27 (27 K) and HumanMethylation450 (450 K) BeadChip datasets, we then systematically investigated DNA methylation profiles of these two sites among normal, LGG and GBM samples. Apparently, the two sites show hypermethylation in GBM patients compared with normal samples (Fig. 4), which is more significant for cg26626089 (Fig. 4a and c; *p-*value < 0.01, Wilcoxon test). Furthermore, we examined the variation of methylation level by using whole-genome bisulfite sequencing data of six GBM samples from TCGA and one normal sample from UCSC (2017 version; http://genome.ucsc.edu, last accessed on 12 May 2019). Consistently, most GBM patients show higher methylation levels than normal samples (Additional file 6: Fig. S5). In addition, considering different-grade gliomas, both sites present much lower methylation levels in GBM samples than LGG samples (Fig. 4e-h; *p-*value < 0.01, Wilcoxon test). We further investigated its methylation in pGBM and rGBM and obtained contradictory results in different populations; *PRKCG* methylation shows no significant difference in the Chinese population (V10-CGGA-M) (Additional file 7: Fig. S6A-B; *p-*value > 0.05, Wilcoxon test) but significantly difference in the Switzerland population (V14-GSE60274-M) [55] (Additional file 7: Fig. S6C-D; *p-*value < 0.05, Wilcoxon test). This is most likely caused by the population genetic difference and/or the small sample size (both datasets have < 5 rGBM samples).

Collectively, *PRKCG* is significantly differentially expressed/methylated among normal, LGG and GBM samples. Compared with normal samples, *PRKCG* presents lower expression and higher methylation in glioma samples. With tumor malignancy, *PRKCG* methylation and expression are both on the decrease (discussed below).

### Combined methylation signatures of *PRKCG* and *MGMT* are more effective in treatment prediction

**Fig. 5:**
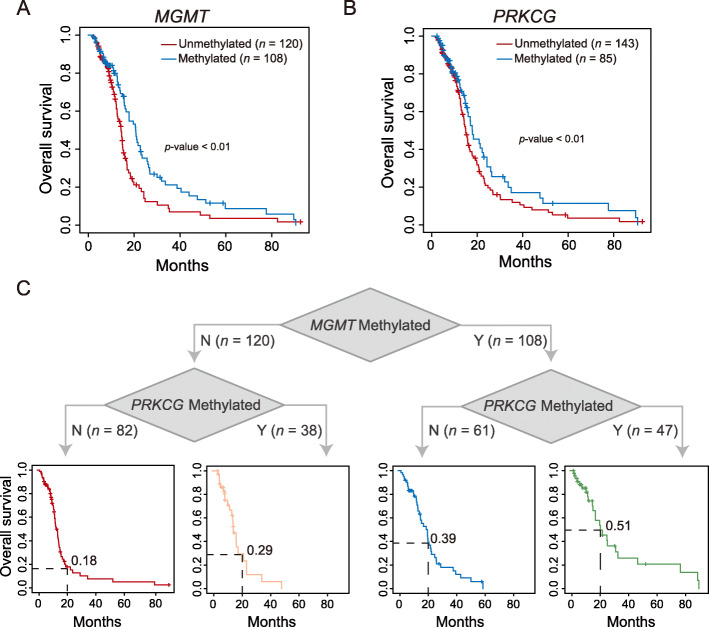
Combined DNA methylation signatures of *MGMT* and *PRKCG* for treatment prediction. **a** Kaplan-Meier survival curves for GBM patients with TMZ treatment based on *MGMT* methylation. **b** Kaplan-Meier survival curves for GBM patients with TMZ treatment based on *PRKCG* (cg26626089) methylation. **c** Kaplan-Meier survival curves for GBM patients with TMZ treatment based on *MGMT* and *PRKCG* combined methylation signatures

It is known that *MGMT* encodes a DNA-repair protein and hypermethylation of *MGMT* is associated with diminished DNA-repair activity, accordingly allowing the alkylating drug temozolomide (TMZ) to have greater effect in GBM treatment [82, 83]. In our study, we obtained consistent results that patients with methylated *MGMT* are more sensitive to TMZ treatment than those with unmethylated *MGMT* (Fig. 5a; *p*-value < 0.01, log-rank test).

Considering that a single molecular biomarker might be lack of sufficient prediction power and thus fail to determine the clinical therapeutic efficacy due to tumor heterogeneity [84], we sought to examine the predictive potential of *PRKCG* for TMZ using 228 GBM samples with matched DNA methylation and clinical data. We discovered that among the two CpG sites of *PRKCG* (cg26626089 and cg04518808), cg26626089 is able to classify patients into two groups with distinct survival advantages, as patients with methylated cg26626089 have significantly longer survival than those with unmethylated cg26626089 (Fig. 5b and Additional file 8: Fig. S7A). By combining *PRKCG* (cg26626089) with *MGMT*, intriguingly, GBM patients receiving TMZ treatment can be classified into four groups that exhibit significantly different survivals (Fig. 5c and Additional file 8: Fig. S7B; *p*-value < 0.01, log-rank test). The four groups, namely, *MGMT*-unmethylated + *PRKCG*-unmethylated, *MGMT*-unmethylated + *PRKCG*-methylated, *MGMT*-methylated + *PRKCG*-unmethylated, and *MGMT*-methylated + *PRKCG*-methylated, present gradually improved longer survivals, as their 20-month OS rates are 0.18, 0.29, 0.39 and 0.51 (Fig. 5c), respectively, implying that the combined methylation signatures of *PRKCG* and *MGMT* might guide more accurate GBM stratification and achieve better personalized therapeutic decisions. Noticeably, elevated *MGMT* expression is associated with TMZ resistance [33]. Similarly, we consistently detected that the both-methylated group with better survival shows significantly lower expression of *MGMT* (Additional file 8: Fig. S7C).

### Multi-omics molecular profiles of *PRKCG*

Based on multi-omics profiles of *PRKCG*, we explored the relationship between *PRKCG* and classical molecular features/glioma grades. First, *PRKCG* is located on the chromosome 19q13.42, unifying previous findings that 1p/19q codel is closely associated with glioma. Consistently, *PRKCG* CNV is closely associated with 19q status (Additional file 9: Fig. S8A and Additional file 10: Fig. S9). Second, *PRKCG* methylation (cg26626089) is associated with *IDH* status, agreeing well with the finding that *IDH*-mut is an initiating event that remodels the glioma methylome, resulting in extensive DNA hypermethylation [12, 85] and thus most likely indicating that *PRKCG* methylation is a passenger of *IDH-*mut status. As LGG samples are always associated with *IDH*-mut and GBM samples are associated with *IDH*-WT, it is not difficult to understand why the methylation level of *PRKCG* is significantly lower in GBM than in LGG (Fig. 4e-h). At the same time, such higher expression level and higher methylation level lead to the suspicion whether *PRKCG* expression in glioma is positively regulated by its DNA methylation or is attributable to its CNV.

As expected, when considering CNV loss and gain separately, *PRKCG* CNV is positively correlated with its expression, as observed in the CNV loss and gain groups, respectively (Additional file 9: Fig. S8B-C; *p*-value < 0.01, Spearman correlation = 0.26/0.32). However, such positive correlation is absent when ignoring the difference of CNV status (Additional file 9: Fig. S8D). According to the dosage effect theory [86], the CNV loss group should not express more *PRKCG* than the CNV gain group. This implies that there is probably another factor rather than CNV to dominantly regulate *PRKCG* expression in glioma. Although it contradicts the commonly accepted negative association between gene expression and promoter CpG methylation, a large-scale pan-cancer analysis has also revealed a positive correlation between promoter CpG methylation and gene expression [87]. Consistently, we did observe significant positive correlations between *PRKCG* expression and CpG methylation within the promoter region (Additional file 9: Fig. S8E-F). This positive regulation of CpG methylation is quite strong, which significantly improves *PRKCG* expression in LGG samples; even these samples exhibit obvious CNV loss (Additional file 9: Fig. S8A). Thus, it is presumably suggested that *PRKCG* is most likely regulated in different ways by DNA methylation, which negatively regulates *PRKCG* expression from normal to tumor but positively regulates the expression within tumor. However, gene expression regulation is a more complicated process involving multiple factors aside from DNA methylation and CNV and more efforts on comprehensive and in-depth molecular characterization of glioma are highly needed to elucidate glioma pathogenesis.

### *PRKCG*-like genes may present heterogeneous roles in glioma tumorigenesis

**Fig. 6:**
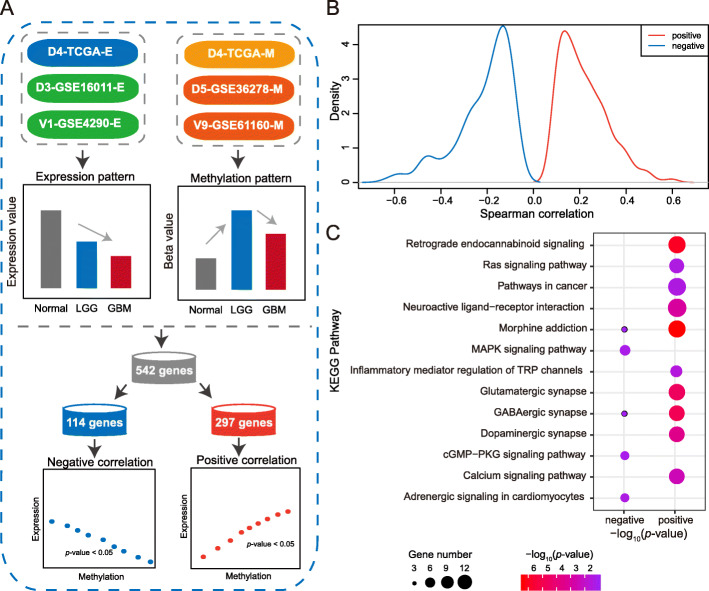
Identification and characterization of *PRKCG*-like genes. **a** Identification of *PRKCG*-like genes, including two types of genes that possess negative and positive correlations between expression and DNA methylation, respectively. **b** Spearman correlations of two types of *PRKCG*-like genes. **c** The KEGG pathway enrichment of two types of *PRKCG*-like genes

To better understand the regulation pattern of *PRKCG*, we further identified a total of 542 *PRKCG*-like genes that possess expression and DNA methylation patterns similar with *PRKCG* (see Materials and Methods) and investigated whether these genes present heterogeneous roles in glioma tumorigenesis (Fig. 6a-b and Additional file 1: Table S3). Noticeably, some of these *PRKCG*-like genes have already been reported to be closely related with glioma [88–92]. For instance, *AKAP6* (A-kinase anchoring protein 6) polymorphisms are associated with glioma susceptibility and prognosis [91]; Phosphorylated *SATB1* (SATB homeobox 1) contributes to the invasive and proliferative of GBM cells and is associated with glioma prognosis [88]; Higher expression of *CDK17* (cyclin dependent kinase 17) is indicative of longer overall survival [89]; *PTPRM* (protein tyrosine phosphatase receptor type M) expression is significantly reduced in GBM by contrast to LGG and higher expression is indicative of longer overall survival [90]; and *CHD5* (chromodomain helicase DNA binding protein 5) might act as a tumor suppressor and its lower expression is associated with poor prognosis in glioma [92]. Among these *PRKCG*-like genes, we revealed that 114 genes show negative correlations between methylation level and expression level, whereas 297 genes present positive correlations (Additional file 1: Table S3). We further performed gene set enrichment analysis for these two groups’ genes. We found that the negatively-correlated genes are primarily enriched in the MAPK and cGMP-PKG signaling pathways, which are essential for tumor cell proliferation and differentiation [93, 94]. On the contrary, the positively-correlated genes are significantly involved in pathways relevant to cancer, inflammation and nerve synapse (Fig. 6c). Thus, *PRKCG*-like genes, presumably present heterogeneous roles in glioma tumorigenesis with complex molecular mechanisms that need further extensive explorations both bioinformatically and experimentally.

## Conclusion

The rapid advancement of sequencing technologies enables large amounts of glioma data generated from different projects and studies throughout the world. Therefore, it has become crucially significant on how to make full use of these valuable data for computational identification and characterization of glioma candidate biomarkers. In this study, we, for the first time, assembled the most comprehensive collection of public glioma datasets with multi-omics data types and different populations/countries. Through comprehensive molecular profiling, we identified that *PRKCG*, a brain-specific gene detectable in CSF, is a potential biomarker for glioma diagnosis, prognosis and treatment prediction. Specifically, it presents lower expression and higher methylation in glioma samples than normal samples. *PRKCG* expression/methylation change from high to low is indicative of glioma progression from low-grade to high-grade and high RNA expression is suggestive of good survival. Importantly, *PRKCG* in combination with *MGMT* is more effective to yield precise survival outcomes after TMZ chemotherapy. In harmony with classical biomarkers, *PRKCG* as well as *PRKCG*-like genes may play important and heterogeneous roles in glioma tumorigenesis. In the era of big data, our findings highlight the importance of computational integrative multi-omics profiling and represent a data-driven scheme toward precision tumor subtyping, accurate therapeutic decisions and better personalized healthcare.

## Supplementary information

**Additional file 1: Table S1.** 15,176 genes’ RNA expression levels across 30 normal human tissues. **Table S2.** Tissue specificity τ values and maximum expression levels of the top 100 brain-specific protein-coding genes. **Table S3.***PRKCG*-like genes and their annotations.

**Additional file 2: Figure S1.** Protein level distributions in CSF and RNA expression profiles of *PRKCG* (A), *BCAN* (B), *OPCML* (C), *GFAP* (D), *CAMK2A* (E) across 30 normal human tissues.

**Additional file 3: Figure S2.** Expression profiles of *PRKCG* (A), *BCAN* (B), *OPCML* (C), *GFAP* (D), and *CAMK2A* (E) across 13 human brain regions.

**Additional file 4: Figure S3.** Expression profiles of *PRKCG* in brain developmental stages.

**Additional file 5: Figure S4.** Expression profiles of *PRKCG* across 31 human tumor and normal tissues.

**Additional file 6: Figure S5.** Bisulfite DNA methylation profiles of *PRKCG* across six GBM samples and one normal sample.

**Additional file 7: Figure S6.** DNA methylation profiles of *PRKCG* in recurrent GBM (rGBM) and primary GBM (pGBM) samples. *PRKCG* methylation profiles were compared between rGBM and pGBM samples (V10-CGGA-M in panels A and B and V14-GSE60274-M in panels C and D). All these datasets can be publicly accessible at ftp://download.big.ac.cn/glioma_data/. The Wilcoxon tests were used and the statistical significance levels were coded by: *ns p* > 0.05, * *p* < 0.05, ** *p* < 0.01 and *** *p* < 0.001.

**Additional file 8: Figure S7.** Predictive potential of *PRKCG* DNA methylation*.* (**A**) Kaplan-Meier survival curves for GBM patients with TMZ treatment based on *PRKCG* (cg04518808) methylation. (**B**) Methylation site cg26626089 in combination with *MGMT*, which were used to classify GBM patients into four groups. (**C**) Expression profiles of *MGMT* in the four groups.

**Additional file 9: Figure S8.** Multi-omics molecular profiles of *PRKCG*. **(A)** Association of *PRKCG*’s multi-omics signatures with *IDH*, 1p/19q status and WHO grade. **(B)** Correlation between *PRKCG* expression and CNV Loss. (**C**) Correlation between *PRKCG* expression and CNV Gain. (**D**) Correlation between *PRKCG* expression and all CNV status. **(E)** Correlation between *PRKCG* expression and DNA methylation of the CpG site cg04518808. **(F)** Correlation between *PRKCG* expression and DNA methylation of the CpG site cg26626089.

**Additional file 10: Figure S9.***PRKCG* CNV associated with survival. **(A)** Four groups of glioma samples were divided based on the 1p/19q status (19q gain, 19q normal, 1p/19q non-codel, and 1p/19q codel). **(B)** Kaplan-Meier survival probability, age, WHO grade and histology of the four groups.

## Data Availability

All datasets integrated in this study were obtained from multiple public database resources (see details in Table 1), which are freely available at ftp://download.big.ac.cn/glioma_data/.

## Abbreviations

CNS: Central nervous system
*IDH*-mut: *IDH* mutation
*IDH*-WT: *IDH* wild-type
WHO: World Health Organization
CSF: Cerebrospinal fluid
TPM: Transcripts Per Million
pGBM: primary GBM
rGBM: recurrent GBM
IT: Infiltrating tumor
CT: Cellular tumor
PAN: Pseudo-palisading cells around necrosis
MVP: Microvascular proliferation
PKC: Protein kinase C
27 K: HumanMethylation27
450 K: HumanMethylation450
TMZ: Temozolomide
