# Supplementary figures and images for: Computational identification and characterization of glioma candidate biomarkers through multi-omics integrative profiling
Source: Biol Direct. 2020 Jun 15;15:10. doi: 10.1186/s13062-020-00264-5 (PMC7294636; doi:10.1186/s13062-020-00264-5)

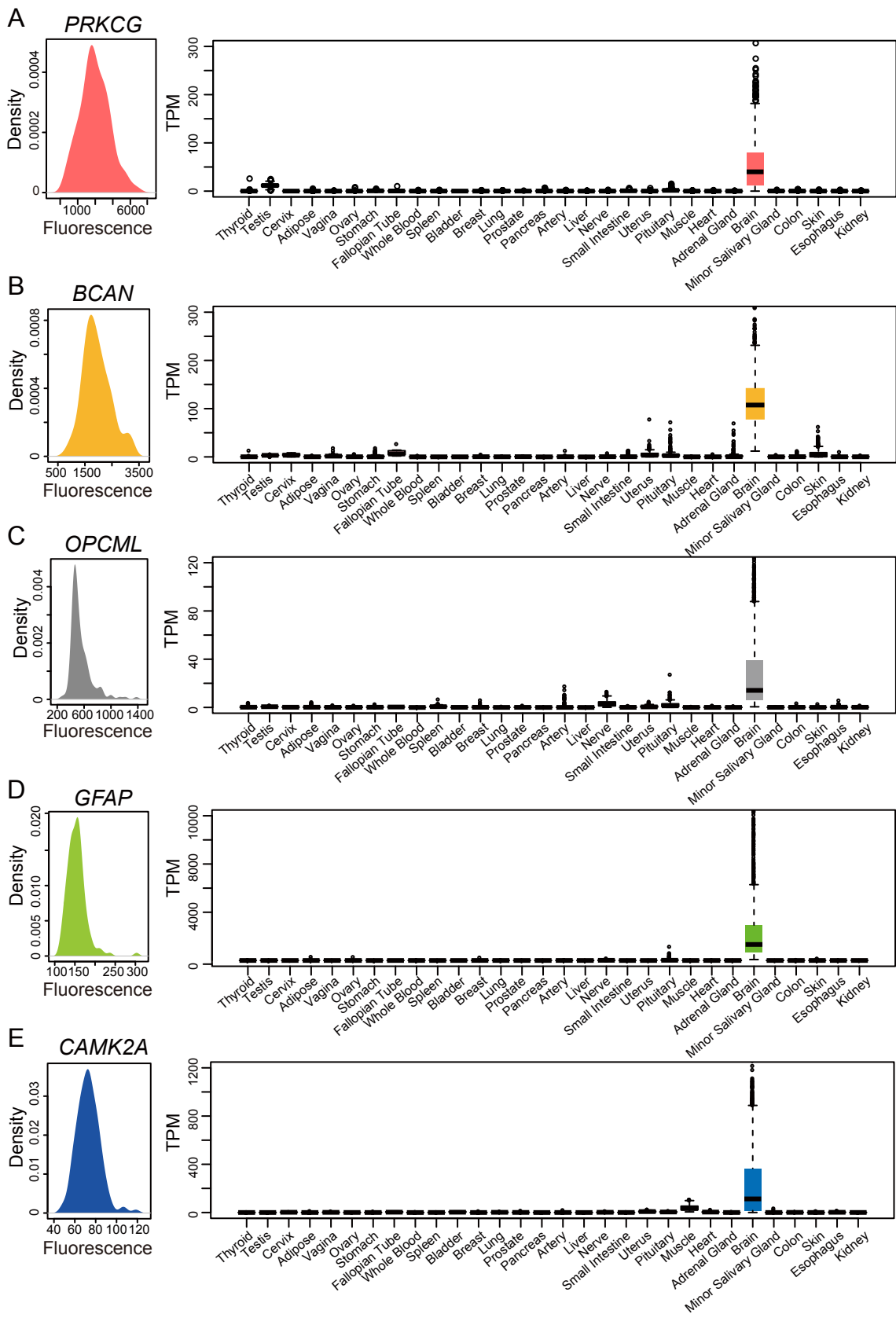

Supplement: Supplementary file 2 — Additional file 2: Figure S1. Protein level distributions in CSF and RNA expression profiles of PRKCG (A), BCAN (B), OPCML (C), GFAP (D), CAMK2A (E) across 30 normal human tissues. [file 13062_2020_264_MOESM2_ESM.pdf]

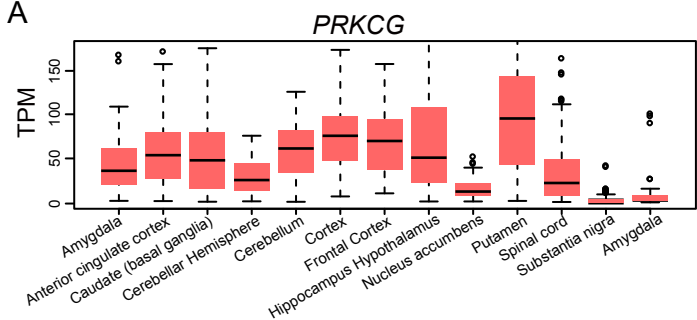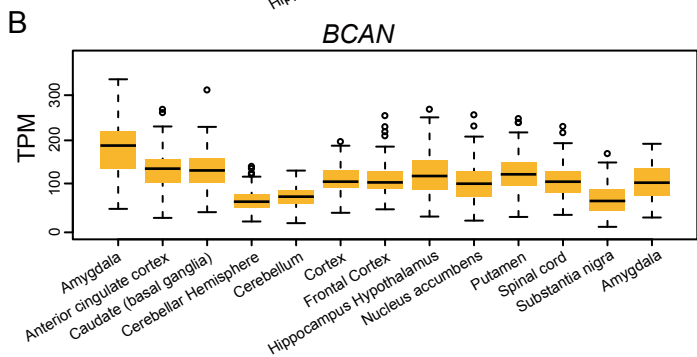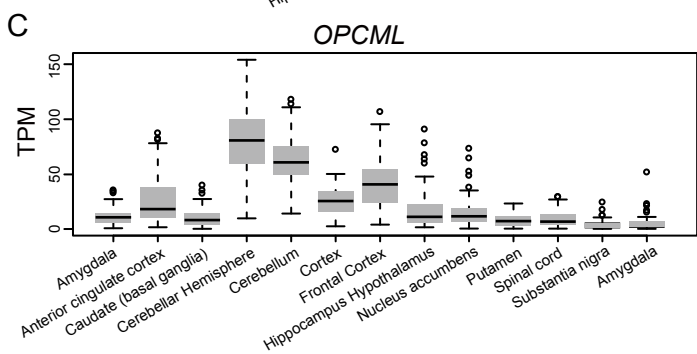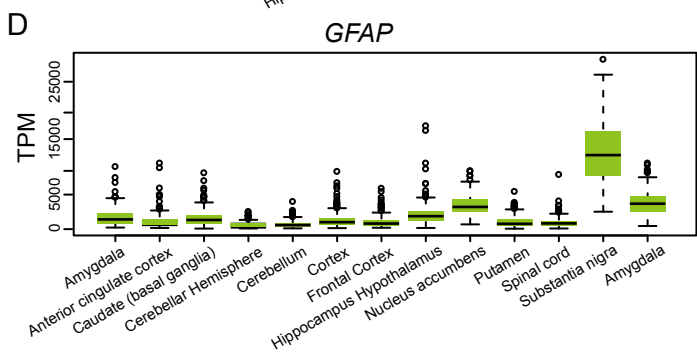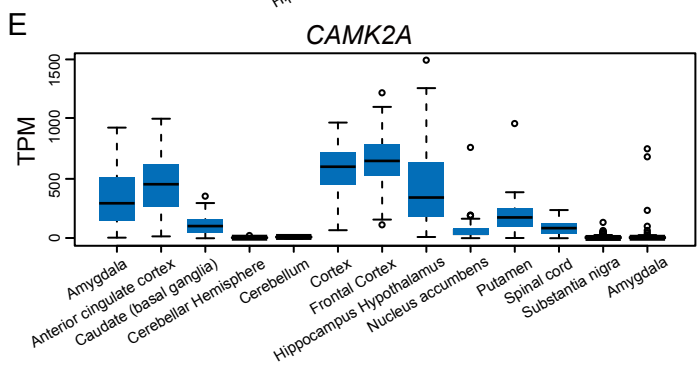

Supplement: Supplementary file 3 — Additional file 3: Figure S2. Expression profiles of PRKCG (A), BCAN (B), OPCML (C), GFAP (D), and CAMK2A (E) across 13 human brain regions. [file 13062_2020_264_MOESM3_ESM.pdf]

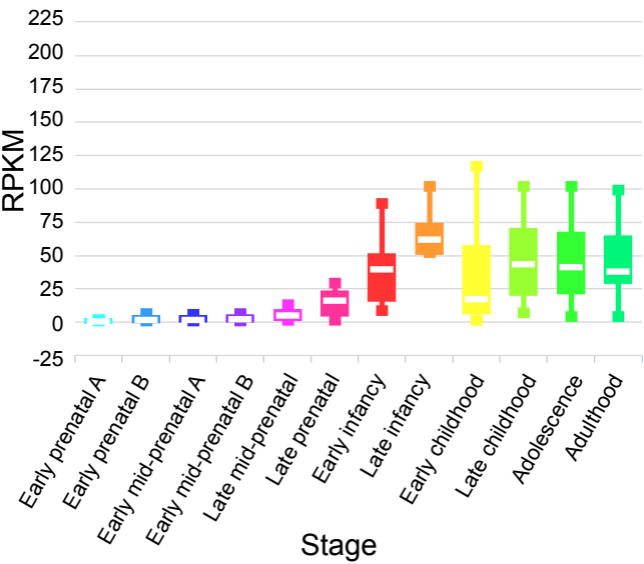

Supplement: Supplementary file 4 — Additional file 4: Figure S3. Expression profiles of PRKCG in brain developmental stages. [file 13062_2020_264_MOESM4_ESM.pdf]

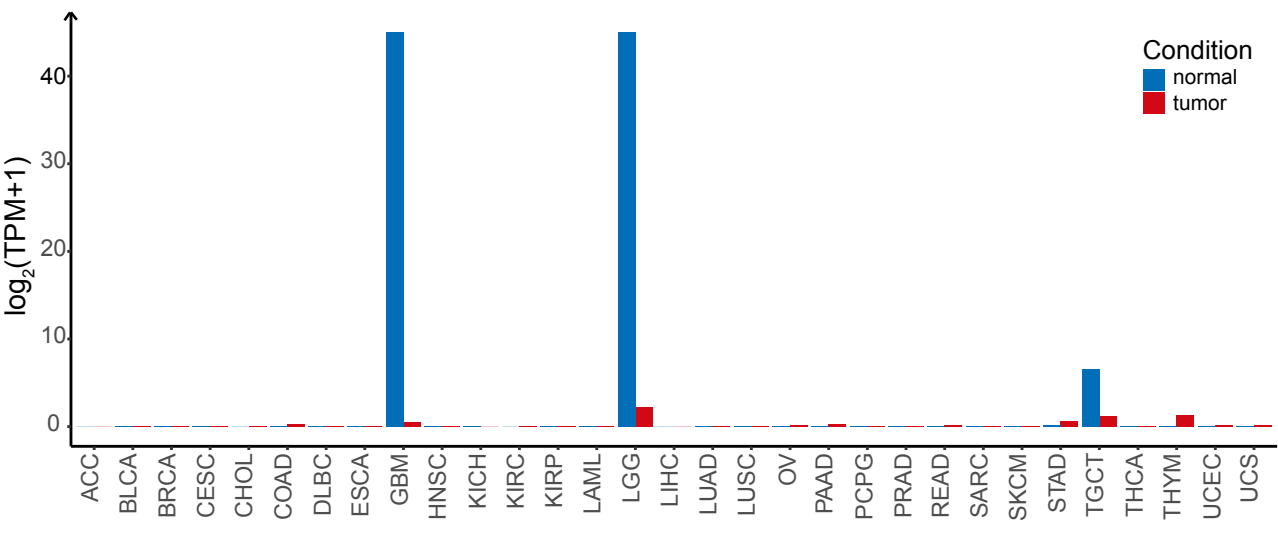

Supplement: Supplementary file 5 — Additional file 5: Figure S4. Expression profiles of PRKCG across 31 human tumor and normal tissues. [file 13062_2020_264_MOESM5_ESM.pdf]

# V11-TCGA-M

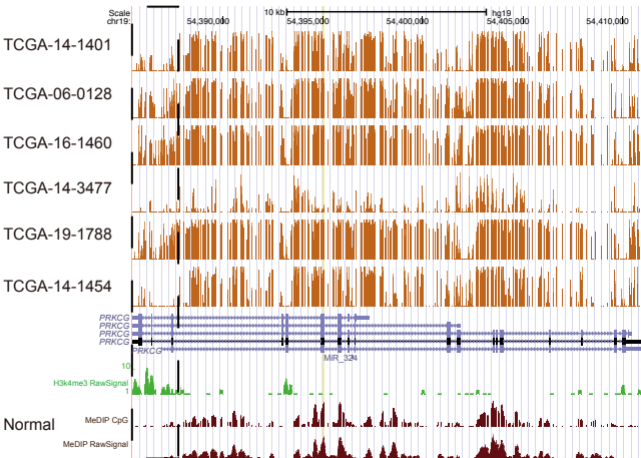

Supplement: Supplementary file 6 — Additional file 6: Figure S5. Bisulfite DNA methylation profiles of PRKCG across six GBM samples and one normal sample. [file 13062_2020_264_MOESM6_ESM.pdf]

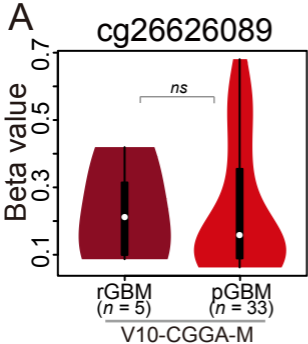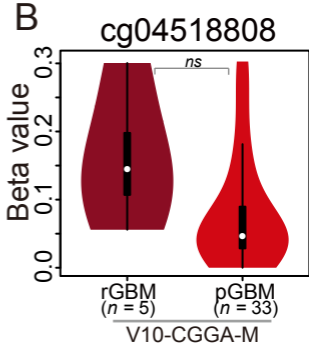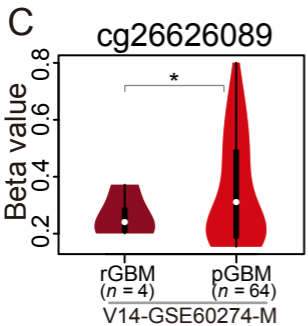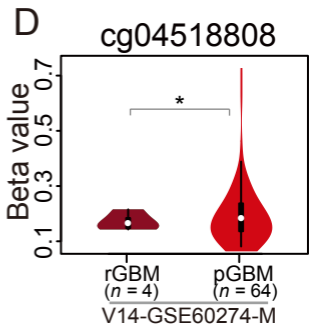

Supplement: Supplementary file 7 — Additional file 7: Figure S6. DNA methylation profiles of PRKCG in recurrent GBM (rGBM) and primary GBM (pGBM) samples. PRKCG methylation profiles were compared between rGBM and pGBM samples (V10-CGGA-M in panels A and B and V14-GSE60274-M in panels C and D). All these datasets can be publicly accessible at ftp://download.big.ac.cn/glioma_data/. The Wilcoxon tests were used and the statistical significance levels were coded by: ns p > 0.05, * p < 0.05, ** p < 0.01 and *** p < 0.001. [file 13062_2020_264_MOESM7_ESM.pdf]

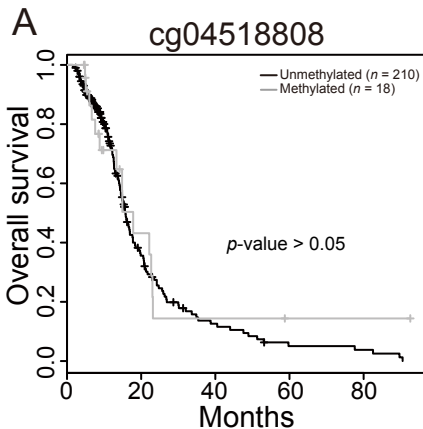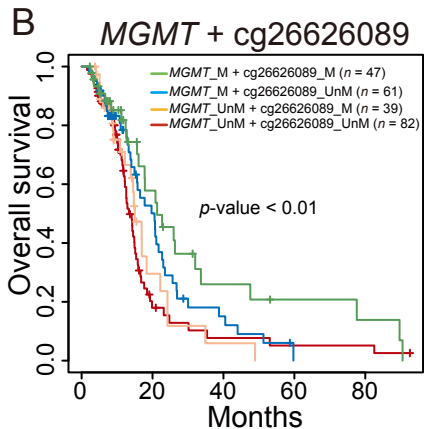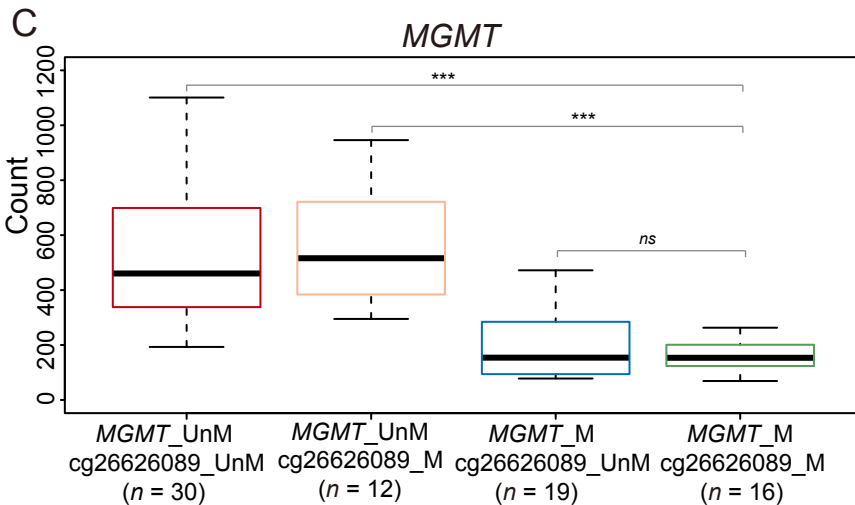

Supplement: Supplementary file 8 — Additional file 8: Figure S7. Predictive potential of PRKCG DNA methylation. (A) Kaplan-Meier survival curves for GBM patients with TMZ treatment based on PRKCG (cg04518808) methylation. (B) Methylation site cg26626089 in combination with MGMT, which were used to classify GBM patients into four groups. (C) Expression profiles of MGMT in the four groups. [file 13062_2020_264_MOESM8_ESM.pdf]

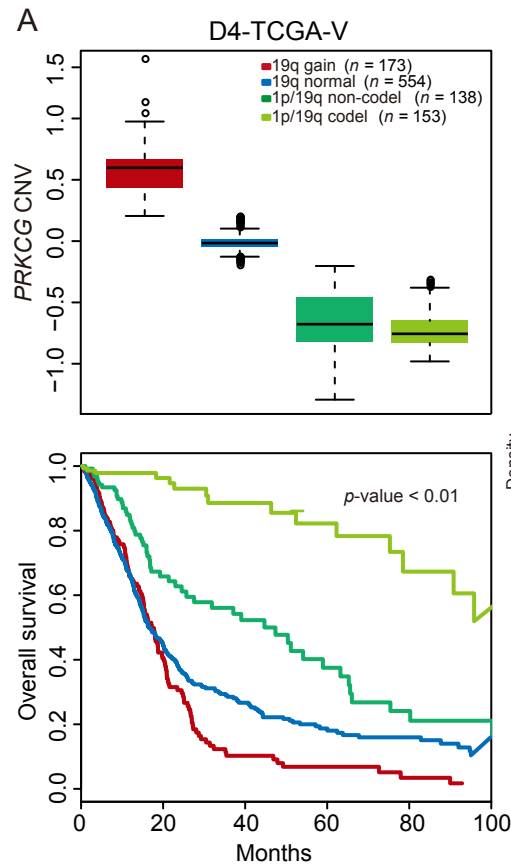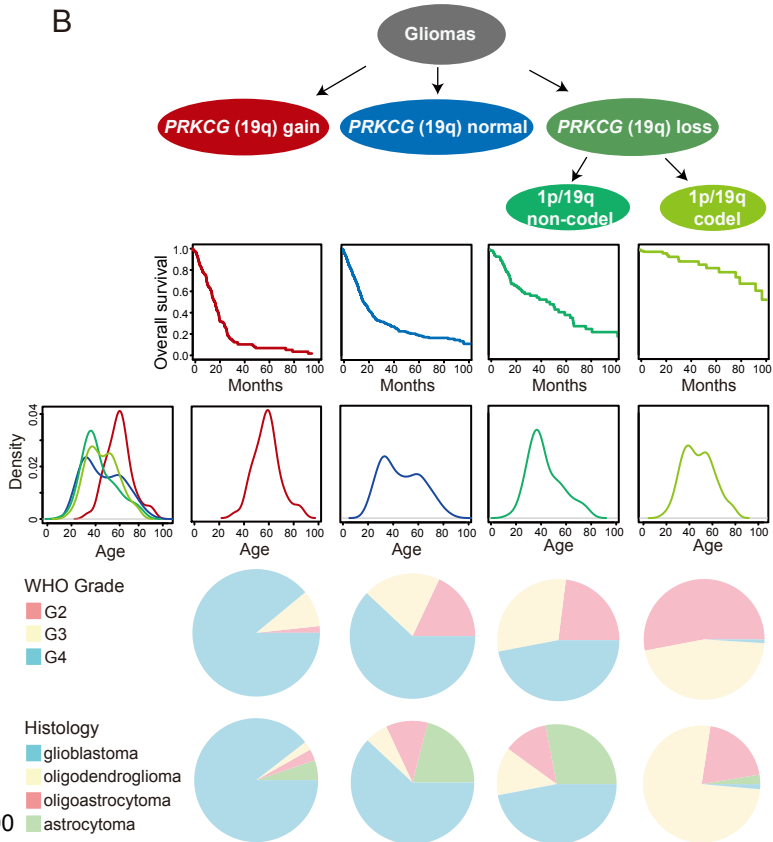

Supplement: Supplementary file 10 — Additional file 10: Figure S9.PRKCG CNV associated with survival. (A) Four groups of glioma samples were divided based on the 1p/19q status (19q gain, 19q normal, 1p/19q non-codel, and 1p/19q codel). (B) Kaplan-Meier survival probability, age, WHO grade and histology of the four groups. [file 13062_2020_264_MOESM10_ESM.pdf]
