# Supplementary material for: Computational identification and characterization of glioma candidate biomarkers through multi-omics integrative profiling
Source: Biol Direct. 2020 Jun 15;15:10. doi: 10.1186/s13062-020-00264-5 (PMC7294636; doi:10.1186/s13062-020-00264-5)
Supplement: Supplementary file 9 — Additional file 9: Figure S8. Multi-omics molecular profiles of PRKCG. (A) Association of PRKCG’s multi-omics signatures with IDH, 1p/19q status and WHO grade. (B) Correlation between PRKCG expression and CNV Loss. (C) Correlation between PRKCG expression and CNV Gain. (D) Correlation between PRKCG expression and all CNV status. (E) Correlation between PRKCG expression and DNA methylation of the CpG site cg04518808. (F) Correlation between PRKCG expression and DNA methylation of the CpG site cg26626089. [file 13062_2020_264_MOESM9_ESM.pdf]

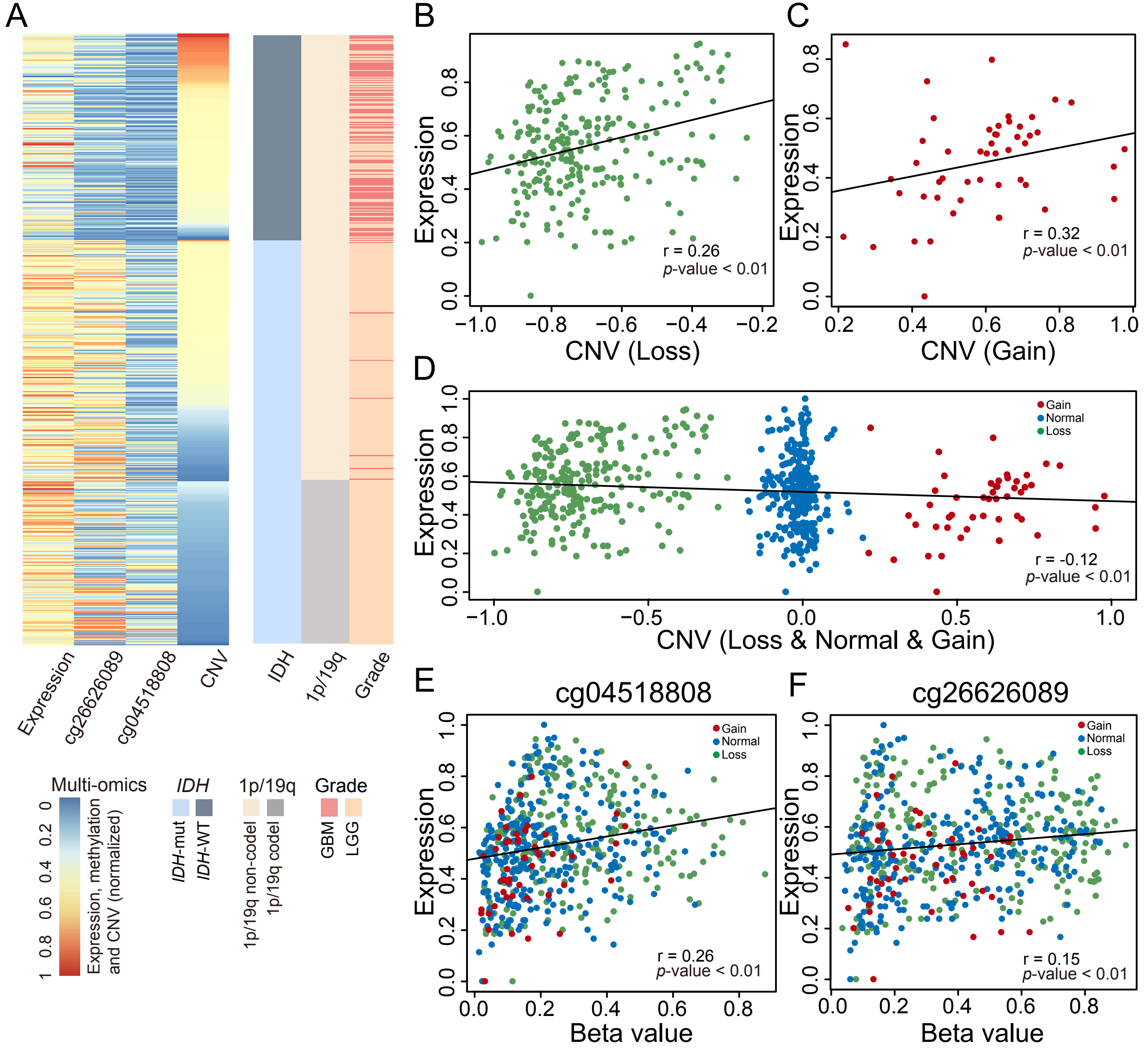
A
B
C
D
E
F
Expression
cg26626089
cg04518808
CNV
IDH
1p/19q
Grade
Multi-omics
Expression, methylation and CNV (normalized)
IDH-mut
IDH-WT
1p/19q non-codel
1p/19q codel
GBM
LGG
CNV (Loss)
r = 0.26
p-value < 0.01
CNV (Gain)
r = 0.32
p-value < 0.01
CNV (Loss & Normal & Gain)
Gain
Normal
Loss
r = -0.12
p-value < 0.01
cg04518808
r = 0.26
p-value < 0.01
cg26626089
r = 0.15
p-value < 0.01
Beta value
